# Notes and News

**Published:** 1899-12-16

**Authors:** 


### Notes and News.

(Collected and Communicated.)

Measles and whooping-cough have been added to the list of diseases which must be notified by physicians practising in Chicago.

In regard to the boasted New York ambulance system we take the following story from the New York *Medical News* for December 2nd: The Harlem Hospital was notified at seven a.m. to hurry an ambulance to 543, East 143rd Street, to attend a woman who had jumped from a third storey window. The ambulance surgeon at once went to that address, but found no patient. On his way back he passed a police station, where he learned that the injured woman was at 543, East 134th Street. He went at once to this place, but an hour's delay had been caused by the mistake, and the patient died soon after his arrival.

The last quarterly court of the governors of the Middlesex Hospital received a report as to the steps which are being taken to render complete and efficient the treatment and study of cancer at the hospital. The new wing for female cancer patients will provide material for the careful observation of the disease. £465 has been voted as an outlay for the special appliances and fittings. Special laboratories have been provided, and a teaching staff of director, assistant director, medical officer, and registrar have been secured. The treasurers of the Middlesex Hospital have received £1,000 from Mr. Arthur W. Davis, with authority for the governors to deal with it as they may think best in assisting the investigation of the subject of cancer.

Some people may have been surprised to find how largely jams and other sugary compounds figure in the feeding of our soldiers. The fact is, that sugar is a food of very considerable value, and is of far greater dietetic importance than would occur to those who look upon it as a mere sweetener, a taste-covering for insipid food. Very elaborate experiments have been carried out in the German army to ascertain how far sugar might be depended on as a food in cases in which considerable exertions had to be made in a short space of time; and it is now considered proved that a sugar diet enables men to put out considerable energy in a comparatively short time, acting in this respect more quickly than some albuminous compounds, although the meal has to be repeated more quickly, as its effect passes off more quickly than is the case with albuminous foods.

The Royal London Ophthalmic Hospital, now fully equipped, was open for the inspection of visitors last week.

At the last quarterly court of the London Hospital it was announced that it was decided to erect an isolation block at the cost of £22,000.

Amongst those appealing for aid for those occupied in carrying on the war in South Africa is Miss Agnes Weston, who has done so much for English sailors. She asks for Christmas or New Year's cheer for the Naval Brigade, and will receive contributions at the Royal Sailor's Rest, Portsmouth.

The first of the military invalids to arrive from South Africa are now installed at the Herbert Hospital, Woolwich. These patients number no wounded amongst them, but are those who have either become invalided before going to the front, or those who have to return thence after becoming incapacitated. The Herbert Hospital holds 600 beds, and Netley is yet larger, but it is to be feared the capacity of both will be taxed to the utmost before the war is over.

With our Special Christmas Supplement, which accompanies this number of The Hospital, our readers will receive two interesting portraits of the Queen. They are respectively titled "The Queen-Empress" and "The Queen-Mother," illustrating as they do the two-fold aspects in which the Sovereign is best known to her people. The portrait of "The Queen-Empress" is reproduced from a photograph taking during her first Jubilee, and the autograph attached is of the same date. This photograph is the only one in which Her Majesty has been taken in her robes of state. The other portrait—"The Queen-Mother"—is a recent one, and so also is the autograph beneath it. These photographs, chosen for reproduction to serve as special supplements to The Hospital, were approved by the Queen for the purpose.

Dr. Robert R. Rentoul proposes that every registered practitioner should be called upon to pay an annual registration fee of £1, the effect of which, he says, would be that the General Medical Council would have an annual income of £34,280, instead of £7,250 as at present. We do not know what Dr. Rentoul would have the General Medical Council do with such a lordly income; but if it is to be the price of the increased direct representation which is the only other plank in his programme, we may question whether the game is worth the candle. When, however, to show the reasonableness of his proposition, he goes on to point out that solicitors pay an annual fee of £6, and auctioneers of £10, we think he is comparing together things which have but little relation to each other. If we as registered practitioners received the same protection from the competition of unregistered persons as solicitors and auctioneers do in their spheres of work, like them we also would be content to pay a fee. But increased direct representation at a cost of over £30,000 a year would, we are afraid, be regarded by most practitioners as a blessing very much in disguise indeed.

